# Shift work is associated with positive COVID-19 status in hospitalised patients

**DOI:** 10.1136/thoraxjnl-2020-216651

**Published:** 2021-03-31

**Authors:** Robert Maidstone, Simon G Anderson, David W Ray, Martin K Rutter, Hannah J Durrington, John F Blaikley

**Affiliations:** 1 NIHR Oxford Biomedical Research Centre, John Radcliffe Hospital, Oxford, UK; 2 Oxford Centre for Diabetes, Endocrinology and Metabolism, University of Oxford, Oxford, UK; 3 The George Alleyne Chronic Disease Research Centre, The University of West Indies at Cave Hill, Bridgetown, Barbados; 4 Faculty of Biology, Medicine and Health, University of Manchester, Manchester, UK; 5 Diabetes, Endocrinology and Metabolism Centre, Manchester University NHS Foundation Trust, Manchester Academic Health Science Centre, Manchester, UK; 6 Department of Respiratory Medicine, Manchester University NHS Foundation Trust, Manchester, UK

**Keywords:** COVID-19, viral infection, occupational lung disease, infection control, respiratory infection

## Abstract

**Introduction:**

Shift work is associated with lung disease and infections. We therefore investigated the impact of shift work on significant COVID-19 illness.

**Methods:**

501 000 UK Biobank participants were linked to secondary care SARS-CoV-2 PCR results from Public Health England. Healthcare worker occupational testing and those without an occupational history were excluded from analysis.

**Results:**

Multivariate logistic regression (age, sex, ethnicity and deprivation index) revealed that irregular shift work (OR 2.42, 95% CI 1.92 to 3.05), permanent shift work (OR 2.5, 95% CI 1.95 to 3.19), day shift work (OR 2.01, 95% CI 1.55 to 2.6), irregular night shift work (OR 3.04, 95% CI 2.37 to 3.9) and permanent night shift work (OR 2.49, 95% CI 1.67 to 3.7) were all associated with positive COVID-19 tests compared with participants that did not perform shift work. This relationship persisted after adding sleep duration, chronotype, premorbid disease, body mass index, alcohol and smoking to the model. The effects of workplace were controlled for in three ways: (1) by adding in work factors (proximity to a colleague combined with estimated disease exposure) to the multivariate model or (2) comparing participants within each job sector (non-essential, essential and healthcare) and (3) comparing shift work and non-shift working colleagues. In all cases, shift work was significantly associated with COVID-19. In 2017, 120 307 UK Biobank participants had their occupational history reprofiled. Using this updated occupational data shift work remained associated with COVID-19 (OR 4.48 (95% CI 1.8 to 11.18).

**Conclusions:**

Shift work is associated with a higher likelihood of in-hospital COVID-19 positivity. This risk could potentially be mitigated via additional workplace precautions or vaccination.

**Key messages**

**What is the key question?**

- Are shift workers in the UK Biobank at higher risk of COVID-19?

**What is the bottom line?**

- Shift workers are more likely to be hospitalised with COVID-19, and this effect cannot be explained by known occupational risk factors.

**Why read on?**

- This risk could potentially be mitigated relatively quickly by increasing distance between workers, wearing personal protective equipment and/or vaccination.

## Introduction

The COVID-19 pandemic has affected millions of people so far. There are limited therapeutic options for COVID-19 causing management to focus on containment.^1^ A greater understanding of risk factors for COVID-19 susceptibility permits protection of the most vulnerable, mitigates occupational exposure and allows for more effective targeting of vaccines.^2 3^ Several risk factors have already been identified for COVID-19 including age, obesity, sex, ethnicity and comorbidities.^2 3^ Occupation has also been recognised as a risk factor for COVID-19 infection with healthcare workers in patient-facing roles being at highest risk.^4–6^ However, the type of working patterns have not been extensively studied despite COVID-19 outbreaks occurring at food-processing factories where night shift workers were employed.^7^

Worldwide shift work is becoming increasingly common with 10%–40% of workers in most countries being involved.^8^ The adverse health effects of shift work are increasingly being recognised. Shift work is associated with respiratory disease,^9 10^ diabetes,^11^ cancer^12^ and non-COVID-19 infectious diseases.^13 14^ The mechanisms underlying these associations remain uncertain; however, sleep disruption, poor diet and circadian misalignment may account for some of the effects.^15^

As the immune system is regulated by the circadian clock, it has been hypothesised that shift work induced circadian misalignment could increase susceptibility to COVID-19 infection.^16^ Current UK guidance from the Health and Safety Executive advocates shift working where possible to limit the number of people in the workplace at any one time.^17^ Therefore, we aimed to investigate the association between shift work status and COVID-19 infection using the UK Biobank.^18^

## Methods

UK Biobank^19^ recruited 502 540 participants (5% of those invited) aged 40–69 years who were registered with the National Health Service and lived within reasonable travelling distance of 22 assessment centres across the UK between 2006 and 2010. During enrolment, they underwent detailed phenotyping including questionnaires administered via touchscreen about employment status and type. Data from the UK Biobank was supplemented through other healthcare resources including the Hospital Episode Statistics, general practitioner (GP) records and other studies using UK Biobank participants.^20^ One of these studies provided updated occupational information from a survey of 120 000 participants investigating occupational work history in 2017.^20^ These data were analysed separately as a subcohort where shift workers who were still working (shift workers 2017) were compared with non-shift workers from the same subcohort.

### Participants

We studied UK Biobank participants after excluding the following groups: (A) healthcare worker testing (defined as reqorg 4, datafield 3311) on the basis that this was not clear whether the healthcare worker was being admitted to hospital with COVID-19 or undergoing routine screening; (B) participants who had COVID-19 testing outside of secondary care; and (C) people who had not provided a detailed job history to determine shift work status.

### Shift work frequency assessment

Shift work was defined as previously reported.^9^ Briefly, participants employed at enrolment in the biobank between 2006 and 2010 were asked to report whether their current main job involved shift work (ie, a schedule falling outside of 09:00 to 17:00). This was 10–14 years before COVID-19 status was assessed. Such schedules involved afternoon, evening or night shifts (or rotating though these shifts). Participants could respond ‘never/rarely’, ‘sometimes’, ‘usually’, ‘always’, ‘prefer not to answer’ and ‘do not know’. For analysis in this study those that answered ‘never/rarely’ were defined as never, those that answered ‘sometimes’ or ‘usually’ were defined as irregular shift workers and those that answered ‘always’ were defined as permanent. If participants recorded the additional options of ‘prefer not to answer’ or ‘do not know’, they were excluded from shift work frequency analysis.

### Shift work type assessment

All participants except those that ‘never’ performed shift work were included in shift work type analysis. They were then asked whether their main job involved night shifts, defined as ‘a work schedule that involves working though the normal sleeping hours, for instance, working though the hours from 12:00am to 6:00am’. Response options were ‘never/rarely’, ‘sometimes’, ‘usually’ or ‘always’ and included additional options: ‘prefer not to answer’ and ‘do not know’. Based on these responses and whether they did shift work, we derived participants’ type of shift work, categorised as ‘none’ (work between hours 09:00 and 17:00), ‘day shift’, ‘irregular night shift work’ (those who answered sometimes or usually) and ‘permanent night shift work’. Participants responding ‘prefer not to answer’ or ‘do not know’ were excluded from this analysis. A subgroup of participants provided updated occupational information in a survey in 2017.^20^ These participants were analysed as current shift workers (shift workers 2017) due to the small numbers (n=43 878).

### COVID-19 positive case definition

Cases of COVID-19 were defined by a positive PCR for SARS-CoV-2 from nasopharyngeal swabs taken from the 16 March to the 24 August 2020 and recorded by Public Health England (PHE).^21^ We confined analysis to those people with an in-hospital PCR test.

### Chronotype

Participants self-reported chronotype on a touchscreen questionnaire at baseline by answering the question: ‘Do you consider yourself to be….’ with response options ‘Definitely a “morning” person’, ‘More a “morning” than “evening” person’, ‘More an “evening” than a “morning” person’, ‘Definitely an “evening” person’, ‘Do not know’ and ‘Prefer not to answer’. Subjects who responded ‘Do not know’ or ‘Prefer not to answer’ were set to missing. This single item has been shown to correlate with sleep timing and dim light melatonin onset. For our analyses we combined ‘more a “morning” than “evening” person’ with ‘more an “evening” than “morning” person’ to form an intermediate group.

### Job sector of shift workers

The OR of COVID-19 was analysed in relation to shift work status by job sector. Job sectors were categorised as ‘essential’, ‘non-essential’ or healthcare worker, as has previously been described.^5^

### Occupation ‘proximity score’

The average physical distance between two individuals employed in particular occupations has been estimated by the ‘proximity score’. We obtained these scores from the Office for National Statistics (ONS)^6^ O*NET database based on workers responses to a question ‘how physically close to other people are you when you perform your current job?’. The answer was then scaled out of 100 and mapped onto the four-digit Standard Occupational Classification (SOC) available for UK Biobank participants.

### Occupation ‘exposure score’

The exposure score is a measure of the exposure of an individual to a disease.^6^ We also obtained these data from the ONS^6^ based on responses to a question ‘How often does your current job require that you be exposed to diseases or infection?’. The answer was scaled out of 100 and mapped onto a four-digit SOC in UK Biobank participants.

### Work environment score

The work environment score was defined as the sum of the proximity and exposure scores.

### Statistical analysis

We employed a multivariate logistic regression model to the data and used this to estimate adjusted ORs and 95% asymptotic CIs on those ORs. Covariates were defined using data collected at the time of enrolment into the UK Biobank. In model 1, we initially adjusted for age, sex, ethnicity and Townsend Deprivation Index (TDI). We extend this adjustment in model 2 to additionally include sleep duration. Lastly, model 3 also included smoking history, alcohol history, body mass index (BMI), hypertension, diabetes, chronotype, cardiovascular disease, renal failure, liver disease, asthma and COPD. Intrajob analysis was performed for occupations where one case of COVID-19 occurred in both shift workers as well as non-shift workers, a paired t-test was then used to compare the incidence between groups. An analysis of variance was used when investigating continuous variables and a χ^2^ test for categorical variables. R (V.4.0.2) was used to analyse data. R packages used include: flex table (V.0.5.11), Magritte (V.1.5), officer (V.0.3.14) and tidy verse (V.1.3.0).

### Patient/public involvement

Participants were not involved in the design or analysis of this study.

### Sensitivity analysis

In participants with proximity and exposure data (n=198 061), we undertook sensitivity analyses to account for the addition of *work environment scores* into model 3, by performing additional analyses after further adjustment for this covariate.

## Results

### Demographics

The UK Biobank included 502 450 participants from which we excluded 1086 healthcare workers and 3050 participants who had COVID-19 testing outside of secondary care (online supplemental figure 1). For frequency of shift work analysis 214 377 participants were excluded since they were not in full-time employment or declined to answer, leaving 284 027 participants (online supplemental figure 1A). Of these SOC,) job codes could be matched to 197 790 participants (online supplemental figure 1A). For type of shift work analysis, 214 035 participants were excluded since they were not in full-time employment or declined to answer, leaving 284 389 participants. Of these SOC job codes could be matched to 198 061 participants (online supplemental figure 1B).

10.1136/thoraxjnl-2020-216651.supp1 Supplementary data

### Clinical characteristics

Clinical characteristics are shown in table 1 for shift work frequency and online supplemental table 1 for type of shift work. Shift workers tended to be younger, male, have a higher BMI, smoke more, have a lower alcohol intake, non-white ethnicity and higher levels of deprivation. Furthermore, they were more likely to have comorbid disease.

**Table 1:** Shift work frequency

	Reported shift work frequency			P values
	Never shift workers	Irregular shift work	Permanent shift work	
N	235 135	27 056	21 836	
Age (years)	52.9 (7.12)	52.16 (7.09)	51.44 (6.86)	<0.01
Sex (% male)	46.61	54.83	55.83	<0.01
BMI (kg/m^2^)	27.09 (4.65)	27.91 (4.92)	28.23 (4.98)	<0.01
**Smoker (%)**				<0.01
Never	58.11	53.09	52.97	
Previous	31.89	31.71	30.77	
Current	9.75	14.77	15.92	
Smoking pack-years	19.99 (16)	23.59 (17.97)	24.04 (17.51)	<0.01
Daily alcohol intake (%)	20.46	17.81	12.89	<0.01
Sleep duration (hours)	7.05 (1.03)	6.92 (1.21)	6.81 (1.39)	<0.01
**Chronotype (%)**				<0.01
Morning	23.34	24.51	22.55	
Evening	8.01	9.04	10.97	
**Ethnicity (%)**				<0.01
White British	88.5	82.06	81.77	
White other	6.44	7.27	6.41	
Mixed	0.65	0.93	0.89	
Asian	1.71	3.63	3.54	
Black	1.39	3.26	4.6	
Chinese	0.34	0.62	0.32	
Other	0.69	1.85	2.11	
Weekly work hours	34.24 (13.19)	37.05 (14.77)	37.68 (12.55)	<0.01
Single occupancy (%)	15.63	18.49	18.99	<0.01
Urban area (%)	85.98	89.1	90.42	<0.01
Townsend Index	−2.24 (−3.7 to 0.18)	−1.43 (−3.25 to 1.55)	−1.05 (−3.02 to 1.95)	<0.01
High cholesterol (%)	7.88	8.48	8.89	<0.01
Diabetes (%)	3.22	4.35	4.58	<0.01
Hypertension (%)	20.33	22.25	22.46	<0.01
Depression (%)	4.61	4.84	5.21	<0.01
Cardiovascular disease (%)	2.27	2.74	2.54	<0.01
Impaired renal function (%)	0.09	0.11	0.11	0.52
Defined asthma (%)	4.93	5.05	5.12	0.32
COPD (%)	0.13	0.2	0.2	<0.01
Liver disease (%)	0.53	0.55	0.47	0.58

Demographics by current shift work exposure (n=284 027). Variables are expressed as mean (±SD) or as percentages.

BMI, body mass index.

Within the UK Biobank, 6442 participants had in-hospital COVID-19 testing, with 498 testing positive. Of these, 316 did not work shifts (‘never’ only worked between 09:00 and 17:00), 98 worked irregular shifts and 84 worked permanent shifts.

### Association between shift work frequency and COVID-19

**Figure 1:**
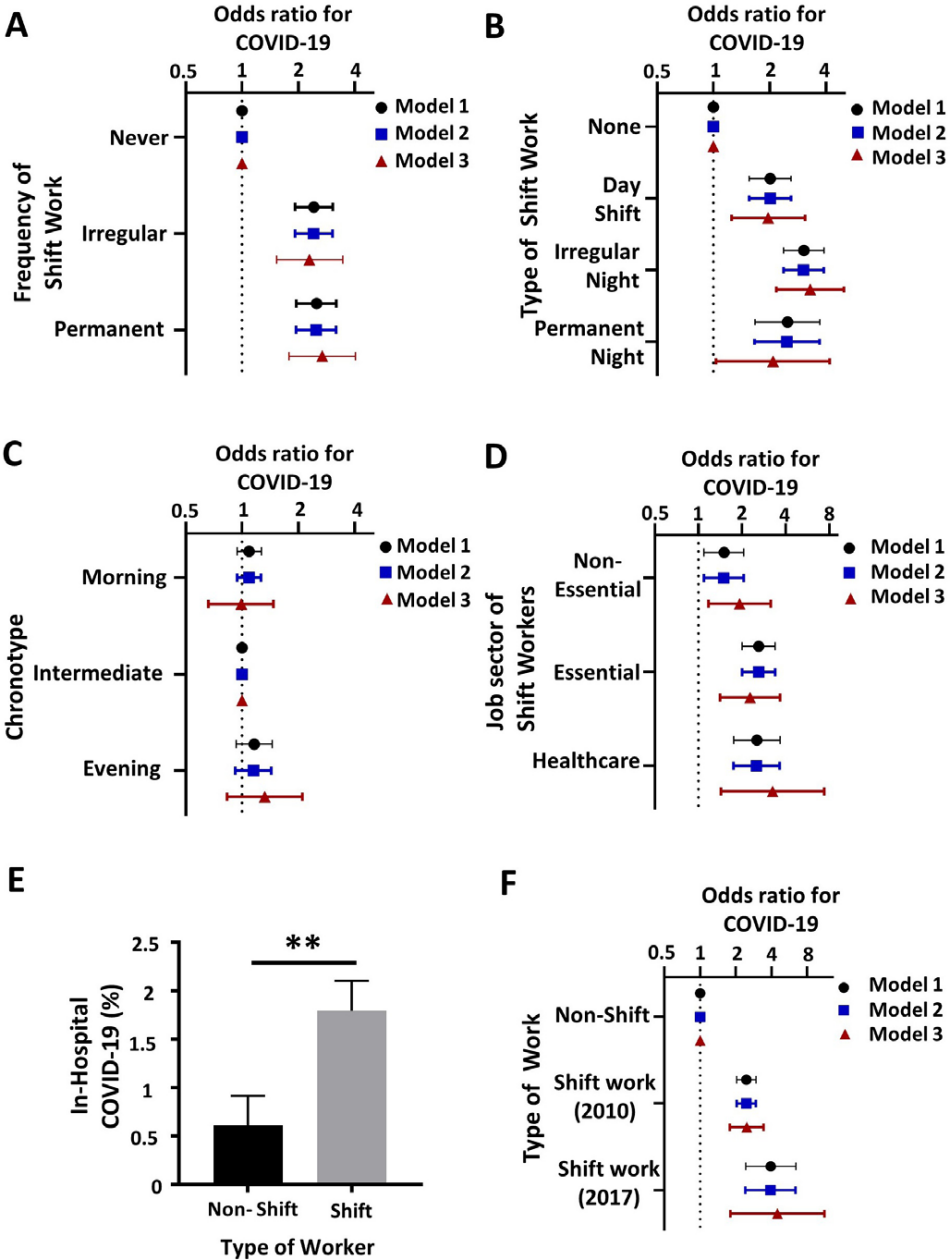
Shift work is associated with COVID-19: workers were stratified by work pattern in the UK Biobank. Figure part A shows the association of shift work frequency with COVID-19. Figure part B shows the association of shift work type with COVID-19. Figure part C shows the association of chronotype with COVID-19. Figure part D shows the association between shift work job sector (non-essential, essential and healthcare worker) and COVID-19. Figure part E shows the difference in COVID-19 frequency between shift workers and non-shift workers who do the same job according to SOC code (n=38 jobs). Figure part F shows the association between shift work status and COVID-19 for those at baseline (‘shift work 2010’) and for those still working when a subgroup of patients were re-evaluated in 2017 (‘shift work 2017’). Model 1 adjusts for the covariates age, sex, Townsend Deprivation Index and ethnicity. Model 2 extended the adjustment to include sleep duration. Model 3 also includes smoking history, alcohol history, BMI, hypertension, diabetes, cardiovascular disease, renal failure, liver disease, asthma and COPD. Chronotype was also included in model 3 for panels A, B D and F. Forrest plots of ORs for COVID-19 with 95% CIs are shown. **=P<0.01, paired t-test (mean±SEM). BMI, body mass index; SOC, standard occupational classification.

To ascertain whether shift work is associated with in-hospital COVID-19 positive test, we compared workers who never worked shifts with participants who worked irregular or permanent shifts. Shift work was associated with a higher likelihood of COVID-19 for both irregular (OR 2.42 (95% CI 1.92 to 3.05)) and permanent shift work (OR 2.50 (95% CI 1.95 to 3.19)) after adjusting for age, sex, ethnicity and TDI (model 1, figure 1A). One of the characteristic features of shift work is sleep disruption and in particular sleep deprivation. After adjustment for sleep duration, the ORs remained broadly unchanged (model 2, figure 1A). As shift work is associated with obesity, smoking, alcohol intake and as chronotype impacts on night shift tolerability, we adjusted for BMI, chronotype, alcohol intake, smoking and prior disease (model 3). The association with irregular shift work remained (OR 2.29 (95% CI 1.53 to 3.45) following this adjustment and increased for permanent shift work OR 2.68 (95% CI 1.78 to 4.03)) (model 3, figure 1A).

### Association between type of shift work and COVID-19

Next, we investigated whether the type of shift work affected the association with COVID-19. Compared with workers who engaged in no shift work (‘none’), day shift workers and night shift workers (working irregular and permanent night shifts) had a higher likelihood of having a positive COVID-19 test after adjustment for age, sex, ethnicity and TDI (figure 1B, model 1). In the same model, irregular night shift work was associated with a higher likelihood of having COVID-19 during hospitalisation (OR 3.04 (95% CI 2.37 to 3.90)), and permanent night shift work was also associated with higher odds (OR 2.49 (95% CI 1.67 to 3.70)). Surprisingly, we also found that workers who worked day shifts also had a higher likelihood of COVID-19 (OR 2.01 (95% CI 1.55 to 2.60)) compared with those reporting no history of shift work. After adjusting for sleep duration, the ORs remained largely unchanged (figure 1B, model 2). Analysis using model 3 also showed a positive association between irregular night shift work and COVID-19 (OR 3.29 (95% CI 2.17 to 4.98)), for permanent night shift workers (OR 2.08 (95% CI 1.03 to 4.18)) and for day shift workers (OR 1.96 (95% CI 1.25 to 3.09)) (figure 1B).

### Chronotype and COVID-19

One possible mechanism for the effects of shift work is through circadian misalignment.^9^ Individuals with extreme chronotypes live misaligned even when not shift working. We found no chronotype association with COVID-19 (figure 1C and online supplemental figure 1C).

### Job sector types and COVID-19

COVID-19 risk is higher in essential workers^5^ and healthcare workers^5^ which could potentially explain our findings if shift workers were concentrated in these groups. The effect of shift work stratified by job sector was therefore examined (online supplemental figure 1A). Compared with colleagues who did not work shifts, non-essential shift workers had a higher OR for COVID-19 (model 1: 1.5 (95% CI 1.09 to 2.05, figure 1D)). Both essential shift workers and healthcare shift workers had slightly higher ORs for COVID-19 (OR 2.6 (95% CI 2 to 3.28), 2.53 (95% CI 1.75 to 3.66)) for model 1. Model 2 hardly affected these figures, in a similar manner to that reported above. Model 3 removed any differences between non-essential workers and other working groups for OR of contracting COVID-19 (figure 1D). Model 3 increased the OR for COVID-19 in non-essential shift workers to 1.92 (95% CI 1.17 to 3.15), in contrast the OR for essential workers reduced to 2.27 (95% CI 1.41 to 3.65). The OR for healthcare workers increased to 3.24 (95% CI 1.43 to 7.36,figure 1D); however, the 95% CIs overlapped with the other groups.

### Job characteristics and COVID-19

COVID-19 risk is also associated with job type,^4–6^ possibly mediated via proximity to other workers or exposure to the disease.^6^ There was no correlation between ‘*proximity score*’ and COVID-19 positive tests (r^2^=−0.166, p=0.98; online supplemental figure 2A) or between ‘*exposure score*’ and COVID-19 positive tests (r^2^=0.2386, p=0.09; online supplemental figure 2B). However, there was a positive correlation between work environment score (combined exposure and proximity score) and COVID-19 (online supplemental figure 2C) (r^2^=0.248, p=0.02).

### Sensitivity analyses

Exposure, proximity and work environment scores were all higher in shift workers (day: n=15 442 and night: n=15 610) compared with non-shift workers (‘none’: n=168 617) (online supplemental table 2), suggesting that the type of job may differ between non-shift workers and shift workers. Therefore, we undertook sensitivity analyses to account for the addition of *work environment scores* in model 3.

For frequency of shift work, after adjusting for model 3 covariates and *work environment* both irregular shift workers (n=17 880, OR 1.95 (95% CI 1.12 to 3.39)) and permanent shift workers (n=12 592, OR 2.01 (95% CI 1.1 to 3.69)) had a higher likelihood of COVID-19 when compared with never shift workers (n=167 318) (online supplemental figure 2D). When type of shift work was examined, after adjusting for the same covariates, compared with non-shift workers, there was an association between irregular night shift work (n=11 173, OR 2.59 (95% CI 1.45 to 4.62)) and COVID-19 (online supplemental figure 2E). However, no significant association for day shift workers (n=15 267, OR 1.74 (95% CI 0.92 to 3.26) or permanent night shift workers (n=4303, OR 1.13 (95% CI 0.35 to 3.68)) was found.

We compared COVID-19 positivity in shift workers and non-shift workers who shared the same job type (SOC code). Shift workers had a higher rate of COVID-19 compared with non-shift workers (n=38 jobs, p<0.01 paired t-test) (figure 1E)

### 2017 shift working job status and COVID-19

A total of 120 307 UK Biobank participants were interviewed in 2017 about their current work status^20^ (online supplemental figure 1D). This revealed that 54.3% of shift workers at baseline were still in shift work for their latest job. Since a proportion of UK Biobank participants had retired, this left 33.5% of the original cohort still performing shift work in 2017. The effects of shift work in those that were still working in 2017 compared with non-shift workers was therefore examined. For model 1, the OR for COVID-19 was 3.94 (95% CI 2.42 to 6.41) and were similar for model 2 (OR 3.91 (95% CI 2.4 to 6.37)). For model 3, which included the most covariates the OR increased slightly to 4.48 (95% CI 1.8 to 11.18) (figure 1F).

## Discussion

We now show that shift workers have higher odds of testing positive for COVID-19 in hospital compared with non-shift workers. Both permanent and irregular shift workers (encompassing both day and night shift workers) had increased odds, compared with workers who never worked shifts. When we stratified shift workers into day shift and night shift workers (including permanent and irregular night shifts), we found that the association with COVID-19 hospitalisation remained increased regardless of the time of day of shift. Sensitivity analysis further revealed that in a subgroup of participants a combination of proximity and exposure scores for job type did not explain the association between shift work and COVID-19 positivity.

The size of effect of shift work as a risk factor for COVID-19 is comparable with other reported risk factors for COVID-19 such as being non-white, being most socioeconomically deprived and having a BMI ≥40 kg/m^2^.^3^ Strikingly, compared with the ORs reported for shift work effects in other diseases in the UK Biobank, in this study, the effects of shift work were much bigger,^9–11^ suggesting this is an important risk factor and should be considered in future public health measures. A key difference with shift work compared with most other COVID-19 risk factors is that this risk could be mitigated relatively quickly. Possible solutions are increasing distance between workers, wearing personal protective equipment and enhanced cleaning of the workspace.

One potential explanation for the effect of shift work on COVID-19 hospitalisation is through the mechanism of circadian misalignment. Supporting this hypothesis is the discovery that melatonin, a drug that can entrain circadian rhythmicity, could be protective against COVID-19.^22^ Early chronotypes experience circadian misalignment when working night shifts and find it difficult to adjust, whereas late chronotypes experience similar disruption when working early shifts.^23^ Therefore, we determined if there was an association between chronotype and COVID-19 hospitalisation. However, no such association was observed. The low numbers of COVID-19 cases for each extreme chronotype (n=274 morning, n=94 evening) suggest that this study may have been underpowered to detect a significant difference for a modest effect comparable with the effect sizes for chronotype in other UK Biobank studies.^10^ Repeating this analysis would be helpful if COVID-19 cases continue to rise.

Another possible explanation for our results is that the type of jobs done by shift workers might increase the association with COVID-19. We did this in four ways; first, by excluding healthcare worker testing a priori from analysis. Second, we used data from the ONS regarding worker proximity and disease exposure and were able to match these codes to two-thirds of the occupations listed for UK Biobank participants. After accounting for worker proximity and disease exposure, statistical significance was lost for some exposures, but the strength and direction of effect remained. We believe these observations are explained by reduced power since some of the categories had only 20 positive COVID-19 cases. Third, we performed an intrajob comparison between shift workers and those that did not perform shift work, which showed higher rates of COVID-19 in the shift work group. Finally, we showed that shift work was associated with higher odds for COVID-19 regardless of job sector type. Therefore, confounding by job type is an unlikely explanation for our results. Alternative explanations for the higher rate of COVID-19 in shift workers might include increased occupancy of workspaces over 24 hours for shift workers, reduced time for cleaning between shifts and tiredness resulting in less awareness of health and safety measures.

Recently shift work has been shown to alter how the immune system responds to infection, and several epidemiological studies have identified that shift workers are more prone to infections.^13 14^ Shift work was not included in the International Severe Acute Respiratory and Emerging Infection Consortium (ISARIC) study^24^ and has not been included as a covariate in other large epidemiological studies.^25 26^ The large association reported in this study would suggest that shift work should be included in future epidemiological pandemic protocols, especially since shift work has been linked to a number of health conditions^27^ including diabetes, obesity, cancer, fibrosis and asthma that altered COVID-19 risk for this pandemic.

The strengths of this study are the large number of individuals, >280 000 participants, that were analysed. Participants were also recruited before the pandemic permitting the control, that is, non COVID-19, group to be selected without bias. However, there are weaknesses in our study. Data collected by questionnaire for the UK Biobank and used in this study were recorded a minimum of 10 years before COVID-19, and although some of the data have been updated through hospital episode statistics, it cannot be viewed as a contemporaneous record. This however is likely to cause bias to the null hypothesis rather than an overstatement of the effect. Indeed, when we analysed the participants who were reinterviewed in 2017 about current work pattern, we found that current shift work exposure increased the OR for COVID-19 hospitalisation. Lastly, accounting for collider bias^28^ in analyses on the UK Biobank data is a non-trivial task, and analysis on COVID-19 disease risk is particularly susceptible to this. We hope to have mitigated this by presenting multiple models of differing complexities, as well as a job paired analysis of the effect of shift work (figure 1E). Despite this, it should still be noted that any conclusions drawn here are made in relation to the UK Biobank cohort only and therefore need to be validated in other populations.

We defined COVID-19 as a positive SARS-CoV-2 test taking place in secondary care. This approach has previously been validated^21^ and identifies those individuals with a more severe form of COVID-19, although we acknowledge that a minority of our cohort could have been picked up during hospital screening. This however could result in selection bias; therefore, repeating this analysis when wider testing becomes available would be useful. Despite this, focusing our research on a more severe type of COVID-19 is important as it is this group of patients that should be targeted for vaccination or enhanced infection control if COVID-19 associated mortality is to be reduced.

## Conclusion

We show that there is an increased likelihood of COVID-19 in shift workers that is comparable with known COVID-19 risk factors. We would advocate that shift work is treated as a modifiable risk factor for COVID-19. Sensible precautions in the workplace for shift workers might include increased after-hours training and supervision on safety protocols, increased cleaning schedules, reduced numbers of workers on any one shift, providing personal protective equipment to shift workers and targeting them for early COVID-19 vaccination programmes.

## Data Availability

Data are available on reasonable request.
