# Supplementary material for: Shift work is associated with positive COVID-19 status in hospitalised patients
Source: Thorax. 2021 Mar 31:thoraxjnl-2020-216651. doi: 10.1136/thoraxjnl-2020-216651 (PMC8098298; doi:10.1136/thoraxjnl-2020-216651)
Supplement: Supplementary file 1 [file thoraxjnl-2020-216651supp001.pdf]

Supplement

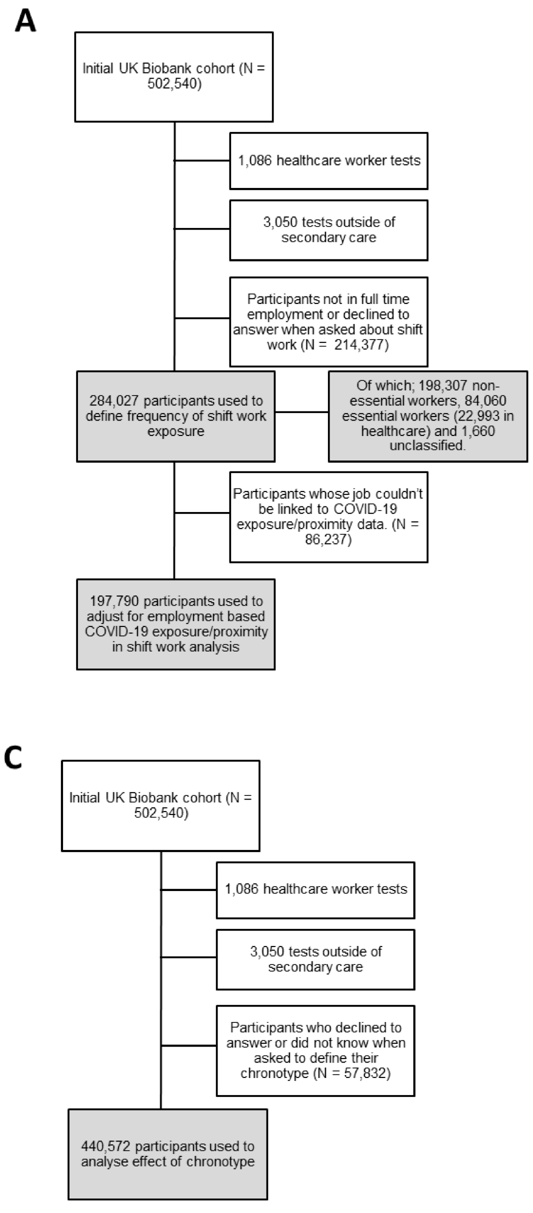

Suppl. Figure 1: Strobe Diagram Participants for this study were analysed in four groups. Shaded boxes show which cohorts were analysed in the paper. **A** Frequency of Shift work and Job Sector **B** Type of shift work **C** Chronotype and **D** 2017 occupational re-profiling

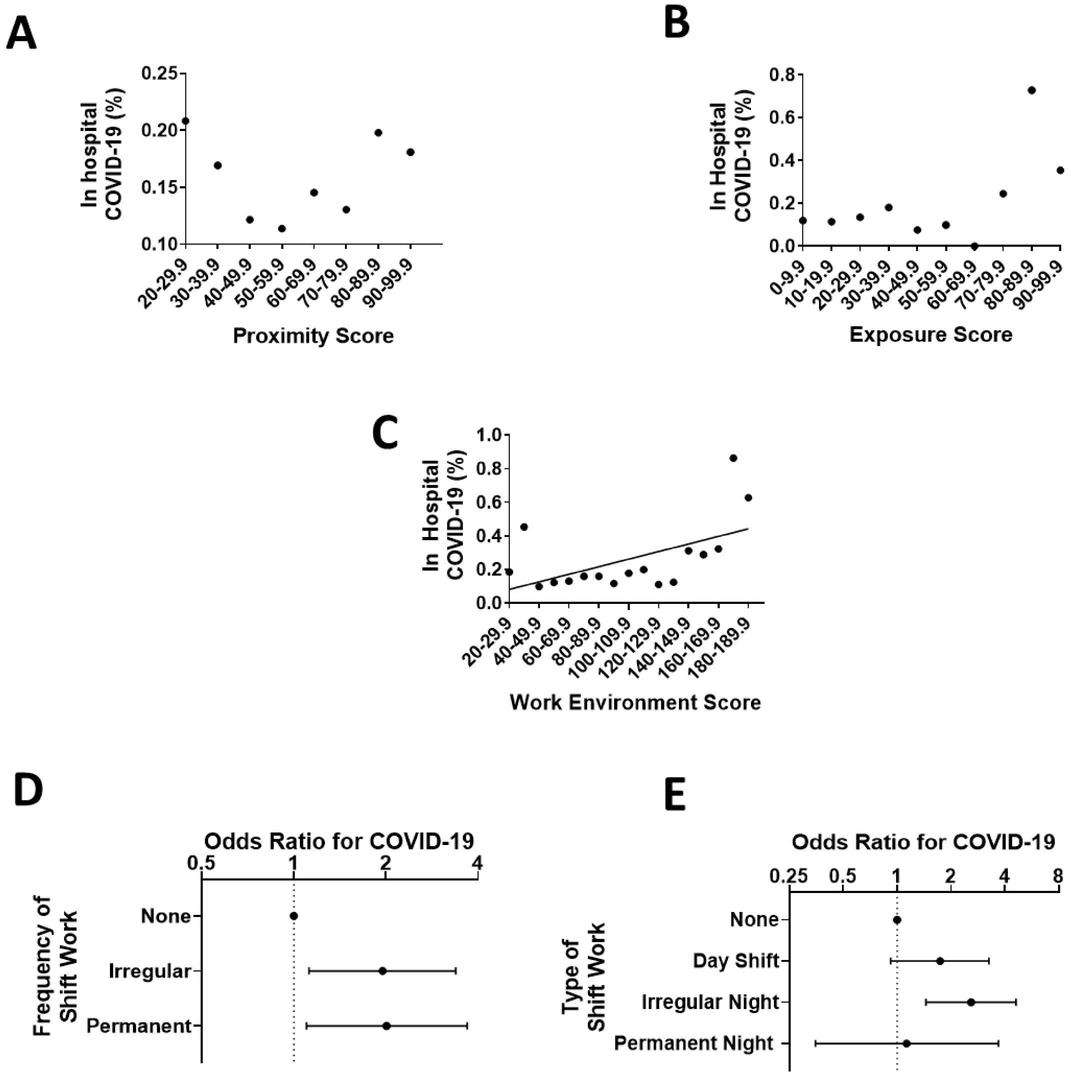

Suppl. Figure 2: Effect of proximity and exposure through occupation on COVID-19 risk. Occupational codes from the UK Biobank were combined with proximity and exposure data from ONS. **A** Risk of COVID-19 as a function of physical proximity. **B** Risk of COVID-19 risk as a function of exposure. **C** Risk of COVID-19 as a function of the sum of proximity and exposure scores (work environment score). **D** Shows the association of frequency of shift work with COVID-19 when the covariates in Model 3 and the work environment score are included in the model **E** Shows the association of the type of shift work with COVID-19 when the covariates in Model 3 and the work environment score are included in the model. Where significant linear regression lines are shown.

	Reported Type of shift work				
	None	Day Shift Workers	Irregular night shift work	Permanent night shift work	P values
N	235135	24245	17971	7038	
Age (years)	52.9 (7.12)	52.5 (7.08)	51.11 (6.87)	51.48 (6.9)	<0.01
Sex (% male)	46.61	47.68	62.76	61.72	<0.01
BMI (kg/m^2)	27.09 (4.65)	27.79 (4.99)	28.22 (4.9)	28.5 (4.87)	<0.01
**Smoker (%)**					<0.01
Never	58.11	53.64	52.73	52	
Previous	31.89	32.08	30.64	30.02	
Current	9.75	13.92	16.15	17.68	
Smoking pack-years	19.99 (16)	22.85 (17.49)	24.23 (17.75)	25.54 (18.32)	<0.01
Daily alcohol intake (%)	20.46	16.95	16	10.2	<0.01
Sleep Duration (h)	7.05 (1.03)	6.95 (1.22)	6.85 (1.3)	6.67 (1.51)	<0.01
**Chronotype (%)**					<0.01
Morning	23.34	25.47	22.91	19.22	
Evening	8.01	7.88	9.84	16.93	
**Ethnicity (%)**					<0.01
White British	88.5	83.35	80.05	81.05	
White Other	6.44	7.08	7.02	6.02	
Mixed	0.65	0.88	0.97	0.88	
Asian	1.71	3.57	3.8	3.42	
Black	1.39	2.66	4.86	5.36	
Chinese	0.34	0.49	0.45	0.67	
Other	0.69	1.61	2.42	2.26	
Weekly work hours	34.24 (13.19)	34.98 (13.21)	39.33 (14.55)	39.62 (13.71)	<0.01
Single Occupancy (%)	15.63	18.76	18.72	18.49	<0.01
Urban area (%)	85.98	89.56	89.33	90.97	<0.01
Townsend Index	-2.24 (-3.7 to 0.18)	-1.31 (-3.18 to 1.6)	-1.25 (-3.17 to 1.8)	-1.04 (-3.02 to 2.06)	<0.01
High Cholesterol (%)	7.88	8.57	8.55	9.29	<0.01
Diabetes (%)	3.22	4.28	4.62	4.62	<0.01
Hypertension (%)	20.33	22.24	22.06	23.26	<0.01
Depression (%)	4.61	5.47	4.52	4.69	<0.01
Cardiovascular Disease (%)	2.27	2.72	2.49	2.9	<0.01
Impaired Renal Function (%)	0.09	0.14	0.08	0.09	0.15
Defined Asthma (%)	4.93	5.28	4.79	5.14	0.06
COPD (%)	0.13	0.23	0.19	0.13	<0.01
Liver Disease (%)	0.53	0.49	0.58	0.5	0.57

Suppl. Table 1: Demographics by type of shift work: (n=284,389) Variables are expressed as mean (±SD) or as percentages. P values show whether there is a significant difference between groups (ANOVA).

	Shift Work Type				
	None	Day Shift Workers	Irregular Night Shift Workers	Permanent night shift workers	P values
N	168,617	15,442	11,270	4,340	
Proximity Score	57.76 (13.1)	62.5 (13.63)	64.68 (13.93)	64.69 (12.3)	<0.01
Exposure Score	17.31 (17.94)	21.37 (19.31)	24.16 (23.59)	22.1 (20.52)	<0.01
Work Environment Score	75.07 (28.05)	83.87 (28.37)	88.83 (34.29)	86.8 (30.12)	<0.01

Suppl. Table 2: Work environment score by type of shift work: (n=198,061) Variables are expressed as mean (±SD). P values show whether there is a significant difference between groups (ANOVA).
